# Wood’s Library of Standard Medical Authors for 1880

**Published:** 1880-05

**Authors:** 


### BIBLIOGRAPHICAL.

Wood's Library of Standard Medical Authors for 1880. In twelve Octavo Volumes, bound in Muslin, and sold by subscription at $15 00.

We are in receipt of four volumes—I, III, IV, and VI, of the series for the present year, as follows:

